# A twelve element dual-band MIMO antenna for 5G smartphones

**DOI:** 10.1371/journal.pone.0288593

**Published:** 2023-12-19

**Authors:** Durria Abbasi, Abdul Aziz, Khaled Aljaloud, Yosef T. Aladadi, Abdul Rehman Chishti, Saad I. Alhuwaimel, Niamat Hussain, Rifaqat Hussain

**Affiliations:** 1 Faculty of Engineering and Technology, The Islamia University of Bahawalpur, Bahawalpur, Punjab, Pakistan; 2 College of Engineering, Muzahimiyah Branch, King Saud University, Riyadh, Saudi Arabia; 3 Department of Electrical Engineering, King Saud University, Riyadh, Saudi Arabia; 4 King Abdulaziz City for Science and Technology, Riyad, Saudi Arabia; 5 Smart Device Engineering, Sejong University, Seoul, Korea; 6 Antenna and Electromagnetics Research Group, School of Electronic Engineering and Computer Science, Queen Mary University of London, London, United Kingdom; Universiti Brunei Darussalam, BRUNEI DARUSSALAM

## Abstract

In this paper, a 12x12 dual-band MIMO antenna for 5G smartphones is proposed. It operates in the sub 6 GHz (2.4GHz and 3.5GHz) frequency bands. The MIMO antenna elements are printed on an FR4 epoxy substrate that has a thickness of 0.8mm. The main substrate measures 150 × 75 × 0.8 *mm*^3^, while the side substrates have dimensions of 75 × 6 × 0.8*mm*^3^. The twelve dual-band antenna elements are compact in size. Each antenna element size is reduced significantly, which is 11.20 × 5.98 *mm*^2^(0.0896λ × 0.04784λ). These antenna elments are arranged in such a way that the MIMO antenna not only provides polarization diversity but also helps in achieving good performance in terms of isolation, which is more than 13.5 dB between two adjacent antenna elements. Another significance of the proposed antenna is that both the frequency bands can be tuned independently by varying the corresponding length of each arm. The performance parameters like efficiency is around 40-56% for the lower band and it is 48-62% for the upper band. The envelope correlation coefficient (ECC) is below 0.04 in both frequency bands for the proposed dual band MIMO antenna.

## 1. Introduction

5G cellular technology is being able to deliver higher data rates and huge traffic size. A Multiple Input Multiple Output (MIMO) antenna system can help to substantially lower data connection latency [1]. A Multiband MIMO antenna system is the potential solution to meet increasing demand of diverse networks with simultaneous access to two or more technologies such as Wi-Fi, GSM, Bluetooth, 4G, and 5G communication systems. A higher order MIMO antenna system may achieve more higher data rates; however it is challenging to arrange large number of antenna elements in a compact size of a smartphone without affecting the isolation between antenna elements. An antenna element with reduced size and multiband characteristics in a higher order MIMO antenna system is needed to achieve required higher data rates and advantage of simultaneous operation for various smartphone applications [2].

Several authors have proposed substantial research works for designing multiband MIMO antennas by using structured monopoles, slot antennas, folded loop antennas, PIFA and dual-polarized antennas for 5G applications. In [3], an 8 element MIMO antenna with a rectangular ground slot has been proposed for 5G smartphones operating in 3.34–3.7 GHz and 4.67–5.08 GHz. This MIMO antenna has an isolation of more than 12 dB, ECC is less than 0.08 and channel capacity is between 35-41 bps/Hz. However, the size of each antenna element is large 16.95 × 17 *mm*^2^. The larger antenna element size is the main limitation to increase order of a MIMO antenna system.

A sub-6-GHz dual-band MIMO quad-antenna system with double shorted loop structure is proposed in [4]. The proposed work comprised four MIMO antenna elements and each of size is 15 × 3 *mm*^2^, which operates in two frequency bands, 3.4-3.8 GHz and 4.8-5 GHz. The isolation between adjacent antenna elements is 10 dB and the ECC value is 0.27, so special attention needs to be paid to reduce mutual coupling between two adjacent antenna elements. A triple band EBG structure based MIMO is proposed in [5]. In this slotted ground plane is applied for achieving good isolation. In [6], two antenna sets of folded loop antennas are integrated, one antenna set of 8 elements operates at 3.5 GHz and another set of 4 antenna elements operates in the 5.2-6 GHz frequency band. The isolation is more than 10 dB for both the bands, however the antenna element size is large, which is 20 × 7 *mm*^2^. A four-element dual-band MIMO antenna operating in 3.4-3.6 GHz and 4.8-4.9 GHz based on quarter-circle-shaped PIFA has been reported in [7]. The ECC value is 0.42 and isolation between neighboring antenna elements is very low about 8.5 dB, which shows that the mutual coupling between antenna elements is little poor. A multiband slot based MIMO antenna with good performance is proposed in [8].

An eight-element dual-band antenna based on folded monopole operating in 3.1-3.8 GHz and 4.8-6 GHz bands has been presented in [9]. The ECC value is below 0.06 and the channel capacity is between 38.5-39.5 bps/Hz for both frequency bands. The antenna element size is 17.85 × 5 *mm*^2^, which is large, and the order of the MIMO antenna is also limited to 8. A dual band aperture fed antenna with dielectric resonator is proposed in [10]. A dual mode 10 elements dual-band MIMO antenna operating in 3.3-3.6 GHz and 4.8-5 GHz frequency bands with isolation of more than 12 dB has been presented in [11]. The ECC value is 0.15 and the antenna element size is 10.6 × 5.3 *mm*^2^ which is compact, however to improve isolation, the MIMO antenna elements contain a neutralization line for decoupling, which may limit to achieve higher order MIMO antenna system.

In [12], a 10-element double T-shaped multiband MIMO antenna with good isolation is proposed, which operates in n42, n43, and n46 LTE bands. The channel capacity calculated is around 41 bps, however, the size of the antenna element is 21.9 × 3 *mm*^2^. An 8-element MIMO antenna comprised of a U-shaped folded monopole antenna and L-shaped slot is designed in [13]. The antenna array is operating in 3.1-3.6 GHz and 5.1-5.9 GHz frequency bands. The antenna element dimension is 14.9 × 4.6 *mm*^2^ with an L-shaped slot of dimension 10.2 × 4 *mm*^2^. Its ECC value is below 0.1 and isolation of more than 12 dB is being reported, however the order of the antenna is restricted to 8 and channel capacity is 38.8 bps/Hz and 39.7 bps/Hz for lower and upper band, respectively.

In [14], a triple band MIMO antenna has been reported, which consists of an array of 8 open slot elements. The operating frequency bands are 3.3-3.8 GHz, 4.8-5 GHz, and 5.1-5.9 GHz. The size of the antenna element is 15 × 3 *mm*^2^ with a ground clearance of 13 × 2 *mm*^2^. Its ECC value is below 0.12 and the channel capacity of the proposed MIMO antenna is between 34.9-37.6 bps/Hz in entire operating bands. The number of the antenna elements is limited to 8 and isolation is 10.5 dB. The mutual coupling between antenna elements needs improvement. Some other MIMO antenna systems are also reported in [15–20], however, there is still need to achieve higher order multiband MIMO antenna system to achieve more higher data rates for smartphones.

In this work a 12 element dual band MIMO antenna with compact size of 11.2 × 5.98 *mm*^2^ (0.0896λ × 0.04784λ)is proposed that operates in 2.4 GHz and 3.5 GHz frequency bands for 5G smartphones. In the proposed work, a meandered structure with a small slit is used to achieve dual band characteristics. Each antenna element is separately fed by a T-shaped feeding structure. It achieves isolation greater than 13.5 dB. Its reflection coefficients are less than -17dB in both frequency bands and the peak channel capacity is 48.3 and 51 bps/Hz for the lower and upper frequency band, respectively.

## 2. Dual-band antenna element design

**Fig 1:**
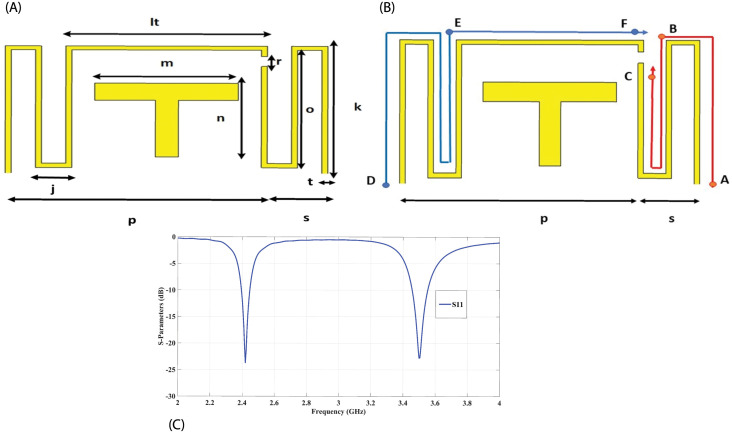
(A) Geometry of Single Element, (B) Arm segments, (C) Reflection coefficient for the single antenna element.

Fig 1(A) represents the detailed geometry of the proposed dual-band single antenna element. The optimized dimensions for the dual-band antenna element are given in Table 1. The dual-band element is comprised of a meandered structure, which has two arms (right arm segment ABC and left arm segment DEF) with a slit between the two arms as shown in Fig 1(B). Both the right and left arms are used to independently tune 3.5 GHz and 2.4 GHz frequency bands, respectively. The dual band antenna element is fed through a T-shaped feeding structure. The size of the dual-band antenna element is 11.20 × 5.98 × 0.02 *mm*^3^(0.0896λ × 0.04784λ × 0.00016λ). The proposed antenna has the advantage of a small size and simple design structure for fabrication and is easily tuneable. Fig 1(C), shows the simulated reflection coefficient for the dual-band antenna element. It can be seen that the proposed antenna element has sharp resonance notches with good impedance matching at 2.4 GHz and 3.5 GHz.

**Table 1:** Single element geometry parameters of the MIMO antenna.

Parameters	*lt*	*m*	*n*	*r*	*o*
Values (*mm*)	7	5	4.25	0.45	5.5
Parameters	*k*	*t*	*j*	*p*	*s*
Values (*mm*)	5.98	0.2	0.85	9.1	2.1

## 3. Working principle of the dual-band antenna element

**Fig 2:**
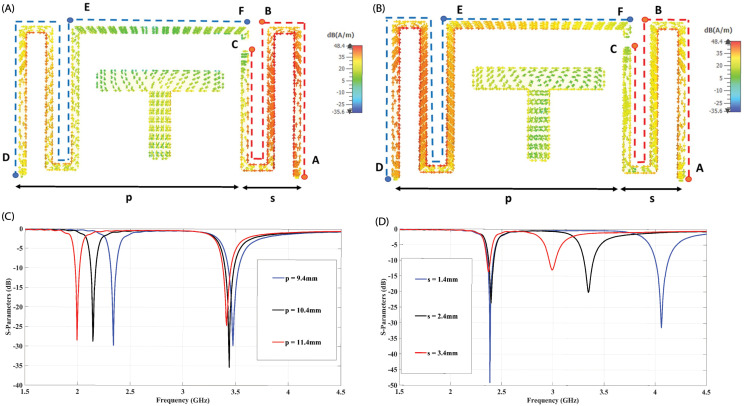
Surface Current Distribution at (A) 3.5 GHz (B) 2.4 GHz. Independent control of resonant frequency (C) varying length of the left arm ‘p’ (D) varying length of the right arm ‘s’.

The working principle of the proposed dual-band antenna element is explained through surface current distributions and parametric analysis in this section. Fig 2(A) and 2(B) show the surface current distributions of the antenna element at 2.4 and 3.5 GHz frequencies, respectively. These will help to understand the basic working principle of the dual-band antenna element. In Fig 2(A), it can be seen that the surface current distribution is significantly more denser at the right arm of the antenna element at 3.5 GHz, which shows that the right arm length is responsible to control the resonant frequency of the higher frequency band, while from Fig 2(B) it can also be observed that the left arm resonates at 2.4 GHz and the significantly strong surface current is following the DEF path in the left arm. Both the left and right arm lengths ‘p’ and ‘s’ are also varied independently to verify independent tuning of both the resonant frequencies and corresponding reflection coefficients are shown in Fig 2(C) and 2(D). It can be seen that the length of the left arm ‘p’ can independently control the lower resonant frequency without affecting the higher resonant frequency, while in a similar way the right arm length ‘s’ is responsible to control the higher resonant frequency, independently.

**Fig 3:**
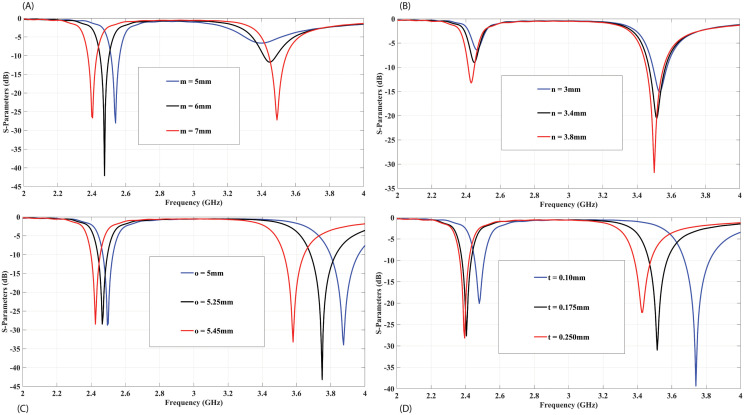
Parametric analysis for the proposed antenna element: (A) varying parameter ‘m’, (B) varying parameter ‘n’, (C) varying parameter ‘o’, (D) varying parameter ‘t’.

The proposed antenna element, a parametric analysis is done to gain a better understanding of its fundamental operating principle, as depicted in Fig 3. The analysis revealed that the parameters ‘o’ and ‘t’ associated with the radiating structure can be adjusted to shift the design frequency to the desired frequency range. Increasing these parameters results in a leftward shift of the resonance frequency, thereby playing a crucial role in reducing the horizontal length of the antenna element. Additionally, the parameters ‘m’ and ‘n’ pertaining to the feed on the backside of the substrate are vital for achieving impedance matching. Modifying these parameters has a notable impact on the impedance matching of the antenna element.

## 4. MIMO antenna structure

**Fig 4:**
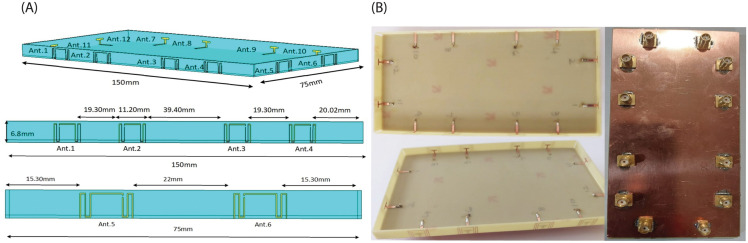
(A) MIMO antenna configuration: longer edgeside view, shorter edge side view (B) Fabricated Prototype of 12x12 Dual-Band MIMO Antenna.

The structure of the proposed 12 element dual band MIMO antenna and its prototype are shown in Fig 4(A) and 4(B), respectively. It can be seen that the eight antenna elements are arranged on both opposite longer sides of the main substrate (four elements on each side) with the same spacing between antenna elements, while two antenna elements are arranged on opposite shorter sides of the main substrate (two elements on each side). Such arrangement of antenna elements also helped to achieve polarization diversity. The main substrate is FR-4 Epoxy with *ϵ*_*r*_ = 4.4, and its dimensions are 150 × 75 × 0.8 *mm*^3^. The longer side edge substrate dimensions are 150 × 6.8 × 0.8*mm*^3^, while shorter side edge substrate dimensions are 75 × 6 × 0.8*mm*^3^. Each antenna element of the proposed design is fed through a 50Ω SMA connector through a hole at the bottom side of the main substrate. The inter-element space between each antenna is 20 mm.

## 5. MIMO antenna performance

For the proposed 12x12 dual band MIMO antenna, the simulation analysis and optimization are performed using CST Microwave studio. The configuration of antenna elements and inter-element spacing has been optimized to achieve the required performance isolation and diversity gain performance in both frequency bands.

**Fig 5:**
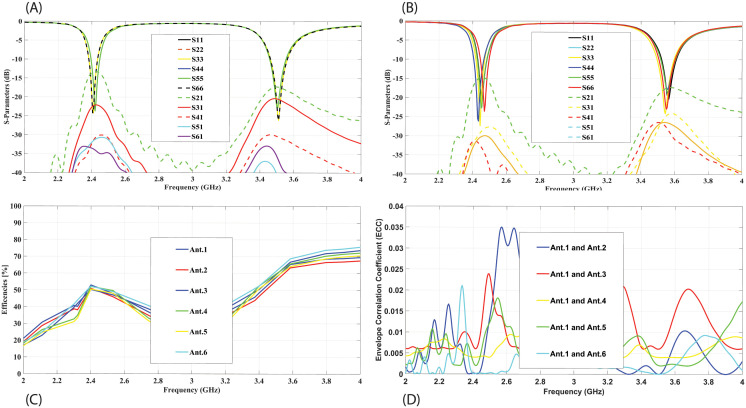
Scattering parameters for the 12x12 MIMO antenna(A) Simulated S-parameters. (B) Measured S-parameters. (C) Efficiencies of Ant.1-Ant6. (D) Envelope Correlation Coefficients (ECC).

The simulated and measured scattering parameters for the proposed MIMO antenna are shown in Fig 5. The simulated curves are shown in Fig 5(A), all the reflection coefficients have good impedance matching in the desired frequency bands of 2.4 and 3.5 GHz, and these are also overlapped very well (for simplicity only S11, S22, S33, S44, S55, and S66 curves are shown). The transmission coefficients curves show that the proposed antenna has isolation better than 13.5dB (for simplicity only S21, S31, S41, S51, and S61 curves are shown).

**Fig 6:**
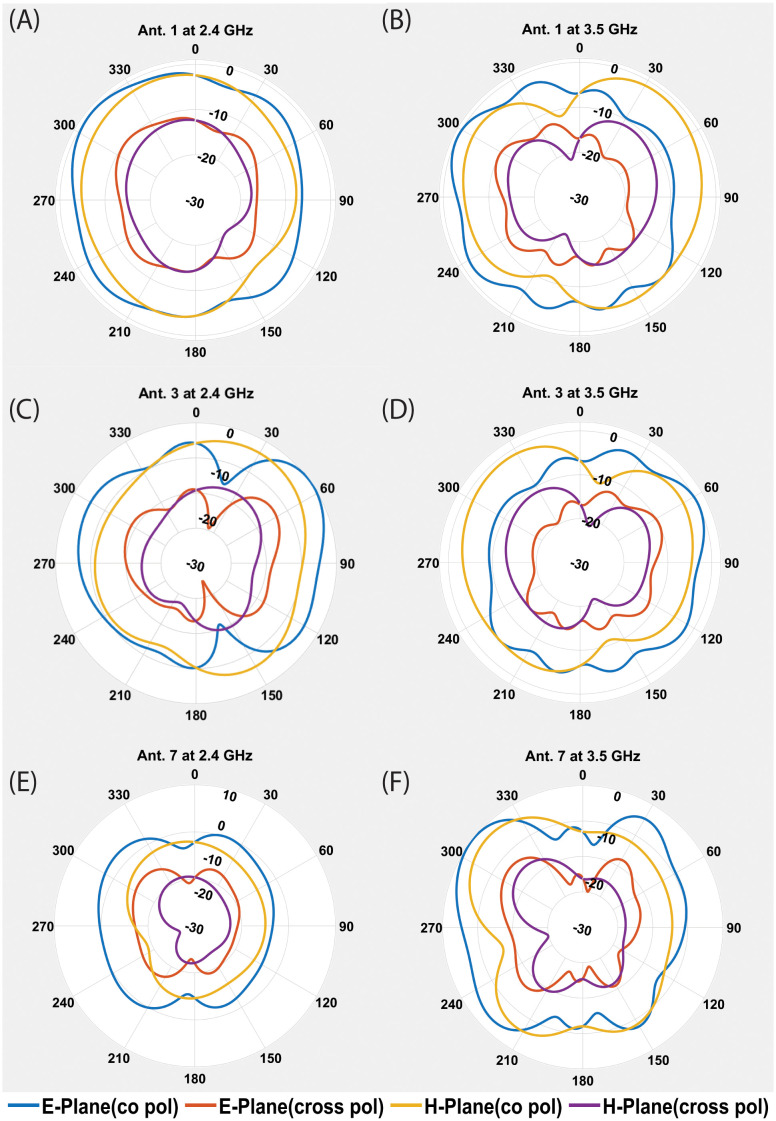
Co-Polarized/Cross Polarized patterns of three antenna elements (A). Ant.1 at 2.4 GHz (B). Ant.1 at 3.5 GHz. (c). Ant.3 at 2.4 GHz (D). Ant.3 at 3.5 GHz. (E). Ant.7 at 2.4 GHz (F). Ant.7 at 3.5 GHz.

**Fig 7:**
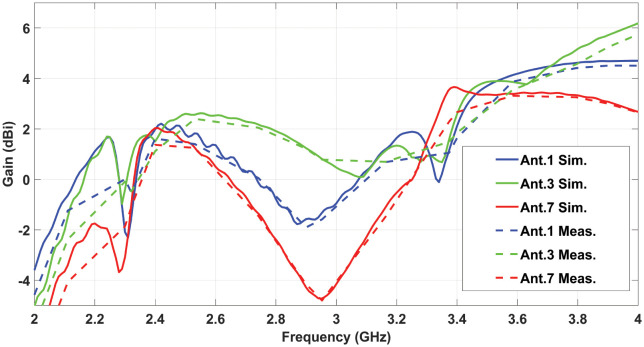
Simulated and measured gains of 12x12 MIMO antenna.

The proposed MIMO antenna’s measured S-parameters are depicted in Fig 5(B). It can be observed in the figure that the measured results are in good agreement with the simulated results. S-parameters shows mutal coupling between antenna element is significantly low. A little shift of 0.01 GHz in 2.4GHz and 3.5GHz resonance frequency towards the right is observed, while measured isolation is little improved, which may be due to fabrication and measurement tolerance. The radiation efficiencies for the first 6 antenna elements are shown in Fig 5(C). It can be seen that the proposed antenna element’s efficiencies have remained between 40% to 56% within the lower frequency band of 2.4 GHz. The radiation efficiency of the MIMO antenna elements in the higher frequency band of 3.5 GHz, lies between 48-62%. In Fig 5(D), the ECC performance for the MIMO antenna system is illustrated. As there is symmetry between the antenna elements, so the ECC performance for only 6 antenna elements is depicted. It can be noted that the ECC value in all the curves is below 0.04 for each antenna pair in both frequency bands. The channel capacity for the proposed 12x12 MIMO antenna is also calculated and it remains between 48.3 to 51 bps/Hz in both frequency bands. Fig 6, illustrated the co-polarized and cross-polarized patterns in both E and H Planes for Antenna1, Antenna 3 and Antenna 7. These three antennas are selected for simplicity due to their unique positions in 12 element configuration of antennas, other all corresponding antennas with similar position have similar performance. It can be seen that the cross polarized pattern have good isolation from co-polarized patterns. The gain over frequency performance of the mentioned antennas is illustrated in Fig 7. The peak gain of three antennas is varied from 1.2 to 1.8 dBi at 2.4 GHz and it is varied from 3.5 to 3.8 dBi at 3.5 GHz.

**Fig 8:**
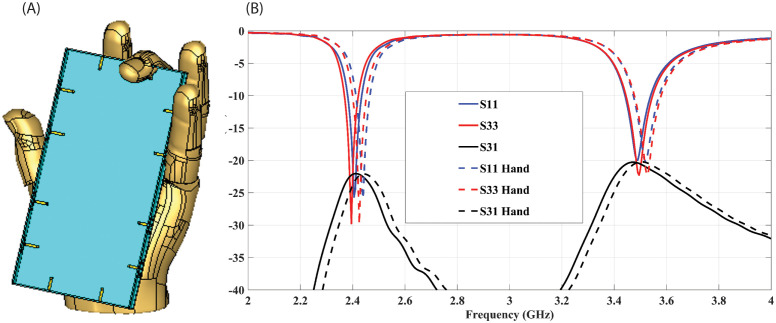
(A) Position of a Single hand on the MIMO antenna. (B) S-parameter of antennas without and with the Hand’s effect.

**Fig 9:**
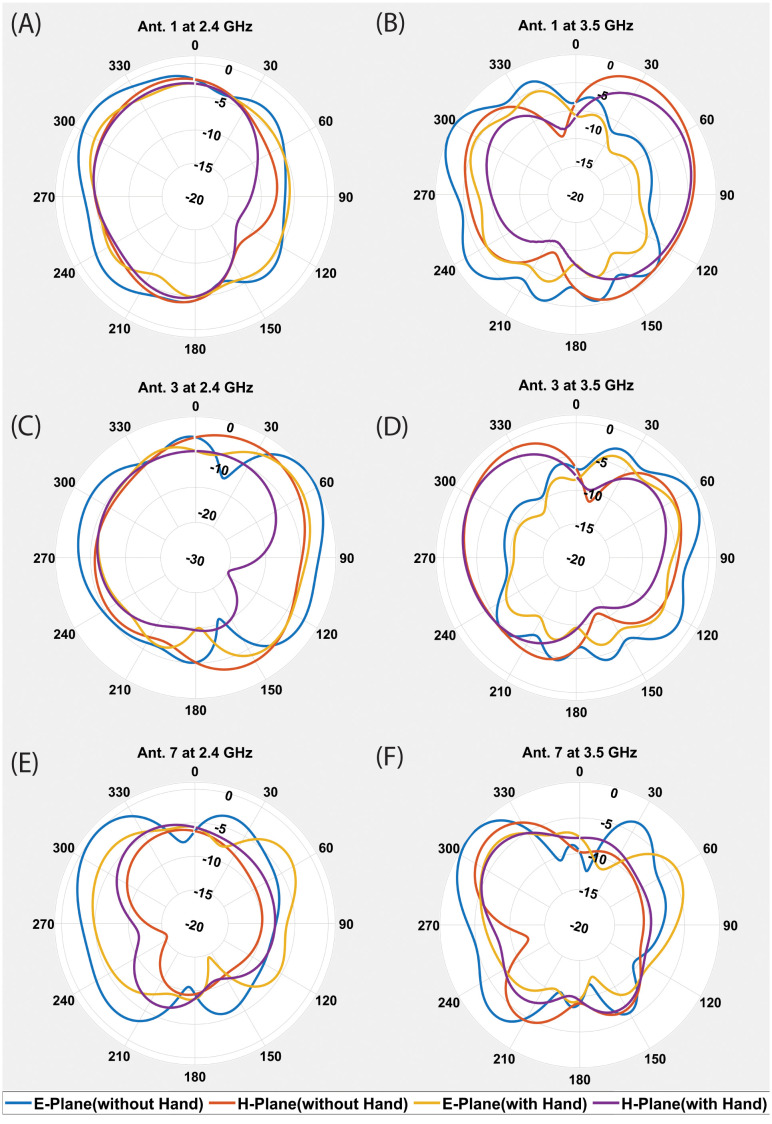
Simulated E and H plane radiation patterns (A). Ant.1 without and with Hand’s effect at 2.4 GHz (B). Ant.1 without and with Hand’s effect at 3.5 GHz. (c). Ant.3 without and with Hand’s effect at 2.4 GHz (D). Ant.3 without and with Hand’s effect at 3.5 GHz. (E). Ant.7 without and with Hand’s effect at 2.4 GHz (F). Ant.7 without and with Hand’s effect at 3.5 GHz.

The proximity of a hand to a Multiple-Input Multiple-Output (MIMO) antenna can result in diverse impacts on its performance. The effect of the user’s hand is examined by incorporating a hand phantom into the simulation. Fig 8 illustrates the effect of single hand on MIMO antenna. Fig 8A depicts the position of MIMO antenna within the hand while Fig 8B presents a comparison of the S-parameter with and without hand’s effect. The results indicate a slight degradation when the MIMO antenna is placed in the hand, along with a minor impact on isolation. In Fig 9A and 9B, the radiation pattern in E and H plane of antenna 1, antenna 3 and antenna 7 at 2.4 GHz and 3.5 GHz is observed to assess the effect of the hand. It can be seen there is degradation in radiation pattern of antenna elements closer to the fingers.

## 6. Comparison with previous works

The Table 2 presented provides a thorough comparison between the proposed MIMO antenna and previous works found in the literature. It is evident that the performance of the proposed MIMO antenna is comparable to that of previous works, with the added advantage of higher order, improved isolation, and enhanced diversity gain performance. However, a minor decrease in efficiency is observed due to significant reduction in antenna size element.

**Table 2:** A comprehensive comparison between the proposed work and other references.

Ref No.	Operating Bands in GHz	Dimensions (λ × λ)	Total Efficiencies	Element No.	Isolation	ECC
[3]	3.34-3.7, 4.67–5.08	0.1697λ × 0.17λ	55%, 72%	8	12	0.08
[4]	3.4-3.8, 4.8-5	0.15λ × 0.03λ	Above 42%	4	10dB	0.27
[9]	3.1-3.8, 4.8-6	0.1785λ × 0.05λ	Above 60%	8	10dB	0.06
[11]	3.3-3.6, 4.8-5	0.103λ × 0.053λ	45-78%, 47-65%	10	12dB	0.15
[13]	3.4-3.6, 5.15-5.925	–	53-56, 53-65	8	12dB	0.1
proposed	2.3-2.45, 3.4-3.6	0.0896λ × 0.04784λ	40-56% 48-62%	12	13.5dB	0.04

## 7. Conclusion

The proposed 12x12 MIMO antenna operates in sub 6 GHz band ranges from 2.3-2.45 GHz and 3.4-3.6 GHz. In addition to this, the center frequency of both the bands can be tuned by varying the corresponding resonant lengths of the antenna element, independently. The dual-band MIMO antenna elements are printed on FR4 epoxy substrate with 0.8mm thickness and the dimensions of the main substrate are 150 × 75 × 0.8 *mm*^3^ while the dimensions of the side substrates are 150 × 6 × 0.8 *mm*^3^ and 75 × 6 × 0.8 *mm*^3^. The antenna elements are self-isolated and the isolation between adjacent antenna elements is more than 13.5 dB. Furthermore, the ECC is below 0.04 with a channel capacity of 48.3-51 bps/Hz in both operating frequency bands.
